# Suspected cases of intracontinental *Burkholderia pseudomallei* sequence type homoplasy resolved using whole-genome sequencing

**DOI:** 10.1099/mgen.0.000139

**Published:** 2017-11-14

**Authors:** Ammar Aziz, Derek S. Sarovich, Tegan M. Harris, Mirjam Kaestli, Evan McRobb, Mark Mayo, Bart J. Currie, Erin P. Price

**Affiliations:** ^1^​ Global and Tropical Health Division, Menzies School of Health Research, Charles Darwin University, Darwin, Australia; ^2^​ Faculty of Science, Health, Education and Engineering, University of the Sunshine Coast, Sippy Downs, Queensland, Australia; ^3^​ Research Institute for the Environment and Livelihoods, Charles Darwin University, Darwin, Australia

**Keywords:** genomics, melioidosis, phylogenetics, homoplasy, source tracing

## Abstract

*Burkholderia pseudomallei* is a Gram-negative environmental bacterium that causes melioidosis, a disease of high mortality in humans and animals. Multilocus sequence typing (MLST) is a popular and portable genotyping method that has been used extensively to characterise the genetic diversity of *B. pseudomallei* populations. MLST has been central to our understanding of the underlying phylogeographical signal present in the *B. pseudomallei* genome, revealing distinct populations on both the intra- and the inter-continental level. However, due to its high recombination rate, it is possible for *B. pseudomallei* isolates to share the same multilocus sequence type (ST) despite being genetically and geographically distinct, with two cases of ‘ST homoplasy’ recently reported between Cambodian and Australian *B. pseudomallei* isolates. This phenomenon can dramatically confound conclusions about melioidosis transmission patterns and source attribution, a critical issue for bacteria such as *B. pseudomallei* that are of concern due to their potential for use as bioweapons. In this study, we used whole-genome sequencing to identify the first reported instances of intracontinental ST homoplasy, which involved ST-722 and ST-804 *B. pseudomallei* isolates separated by large geographical distances. In contrast, a third suspected homoplasy case was shown to be a true long-range (460 km) dispersal event between a remote Australian island and the Australian mainland. Our results show that, whilst a highly useful and portable method, MLST can occasionally lead to erroneous conclusions about isolate origin and disease attribution. In cases where a shared ST is identified between geographically distant locales, whole-genome sequencing should be used to resolve strain origin.

## Abbreviations

BTFC, Burkholderia thailandensis-like flagella and chemotaxis; DPMS, Darwin Prospective Melioidosis Study; ML, maximum-likelihood; MLST, multilocus sequence typing; MP, maximum-parsimony; SNP, single-nucleotide polymorphism; ST, sequence type; WGS, whole-genome sequencing; YLF, Yersinia-like fimbriae.

## Data Summary

1. Whole-genome sequencing data have been deposited in the NCBI Sequence Read Archive (SRA) under BioProject accession number PRJNA393909 (url – https://www.ncbi.nlm.nih.gov/bioproject/PRJNA393909).

2. The SRA accession number for the raw sequence data of MSHR0052 is SRR5818275.

3. The SRA accession number for the raw sequence data of MSHR0116 is SRR5818274.

4. The SRA accession number for the raw sequence data of MSHR3528 is SRR5818273.

5. The SRA accession number for the raw sequence data of MSHR9076 is SRR5818272.

6. The SRA accession number for the raw sequence data of MSHR4608 is SRR5818271.

## Impact Statement

Melioidosis is a high-mortality tropical disease that is acquired following contact with soil or surface waters containing *Burkholderia pseudomallei*. Characterising the spread and genetic diversity of *B. pseudomallei* is critical for understanding melioidosis epidemiology. Multilocus sequence typing (MLST) is a popular genetic tool for fingerprinting bacterial populations. However, the relatively low resolution of this method means that isolates can occasionally be assigned the same sequence type (ST) despite being otherwise unrelated, a phenomenon known as ST homoplasy. Using whole-genome sequencing (WGS), two cases of ST homoplasy occurring between Asian and Australian *B. pseudomallei* isolates have been previously identified. Here, we used WGS to uncover two intracontinental cases of ST homoplasy in isolates from northern Australia, with a third suspected case shown to be a true long-range (460 km) dispersal event. This latter finding points to the relatively recent transmission of a *B. pseudomallei* clone into a new region.

## Introduction

*Burkholderia pseudomallei* is a Gram-negative soil-dwelling bacterium that causes melioidosis, a potentially fatal tropical infectious disease that is hyper-endemic in northern Australia and parts of Southeast Asia [1]. *B. pseudomallei* is an opportunistic organism that infects both humans and animals, although person-to-person and zoonotic transmissions are exceedingly rare [2]. *B. pseudomallei* infections are almost exclusively acquired via contact with a contaminated environmental source [3]. The potential use of *B. pseudomallei* as a biological weapon [4], combined with limited treatment options, difficulty in diagnosis and current lack of a vaccine [1] make this organism of substantial importance to public health in tropical regions worldwide.

Multilocus sequence typing (MLST) [5] is a widely adopted genotyping method for characterising the population diversity of several pathogenic bacterial species, including *B. pseudomallei*. The *B. pseudomallei* MLST scheme [6] uses seven housekeeping loci (*ace*, *gltB*, *gmhD*, *lepA*, *lipA*, *narK* and *ndh*) encoded solely on chromosome 1, and yields sequence data of a combined total length of 3401 bp. In *B. pseudomallei*, MLST has been used extensively to identify strain relatedness [6, 7], for point-source attribution [8, 9] and to document the geographical distribution of isolates, particularly those from the ‘Top End’ region of the Northern Territory, Australia [10, 11]. A major finding to arise from this body of work is that, despite severe weather events (e.g. cyclones, floods), animal migration and anthropogenic factors, *B. pseudomallei* dispersal in the environment is surprisingly limited [10]. Indeed, identical sequence types (STs) are typically found across a maximum linear distance of only 45 km [11].

High rates of genetic recombination [12], coupled with genetic drift over large timescales, can lead to occasional instances where *B. pseudomallei* strains have converged on the same ST by chance rather than by sharing a recent common ancestor. Such cases are termed ST homoplasy [13]. To date, there have been two documented intercontinental cases of ST homoplasy in *B. pseudomallei*, which occurred in isolates retrieved from both Cambodia and Australia [13].

Unlike MLST, whole-genome sequencing (WGS) provides high resolution of closely related isolates and can more accurately characterise the origin of an isolate. Phylogenetic reconstruction of *B. pseudomallei* populations using WGS data is a robust way to identify the geographical origin of strains on a continental level [12–16]. In the present study we use WGS to uncover, for the first time, two intracontinental cases of ST homoplasy in *B. pseudomallei*, with a third suspected case instead being identified as a true long-distance ST dispersal.

## Methods

The ongoing Darwin Prospective Melioidosis Study (DPMS) [17] has documented and collected *B. pseudomallei* isolates from >1000 human melioidosis cases occurring in the ‘Top End’ region of the Northern Territory since October 1989. This collection also includes isolates from animal infections and from environmental sampling efforts undertaken across the Northern Territory since 1991. Most DPMS isolates have previously been genotyped using a combination of conventional MLST [6] or MLST derived from WGS [18]. GPS data were collected at the time of sampling for the environmental isolates, whereas the provenance of clinical isolates was based on patient travel history taken at the time of diagnosis. Using this dataset, isolates belonging to the same ST that were separated by a linear distance of >100 km were investigated as potential cases of intracontinental ST homoplasy.

Comparative analysis of 145 publicly available *B. pseudomallei* genomes that represent a global snapshot of isolates [15], plus five new genomes sequenced as part of this study, was carried out to determine the genetic relatedness of the suspected ST homoplasy cases. ST-149, ST-722 or ST-804 isolates (MSHR0052, MSHR0116, MSHR3528, MSHR9076 and MSHR4608) that lacked existing WGS data were sequenced using the Illumina HiSeq2500 platform (Australian Genome Research Facility). Publicly available data belonging to previously sequenced ST-149 isolates (MSHR0503, MSHR0356 and MSHR4300) were retrieved from the NCBI SRA database. Orthologous, biallelic single-nucleotide polymorphisms (SNPs) were identified from the Illumina data using the default settings of SPANDx v3.2 [19]. The closed Australian *B. pseudomallei* genome MSHR1153 [20] was used as the reference for read mapping for the global phylogeny, whereas the environmental ST-149 isolate MSHR4300 [20] was chosen as the reference for examining ST-149 diversity as this genome is a high-quality assembly, it has the fewest number of contigs (*n*=3) and it has excellent provenance due to its environmental origin. A draft assembly of an additional ST-149 isolate, MSHR0503, was assembled using the Microbial Genome Assembler Pipeline (MGAP) v1.0 [21]. Both maximum-parsimony (MP) and maximum-likelihood (ML) phylogenetic trees were reconstructed based on 207 694 SNPs identified among the 150 genomes using PAUP* 4.0a151 [22] and RAxML v8.0.X (GTRCAT model) [23]. Recombinogenic SNPs were identified using Gubbins v2.2.0 (default parameters) [24]. *In silico* MLST was performed using the BIGSdb tool [25], which is available on the *B. pseudomallei* MLST website (http://pubmlst.org/bpseudomallei/).

## Results

**Fig. 1.**
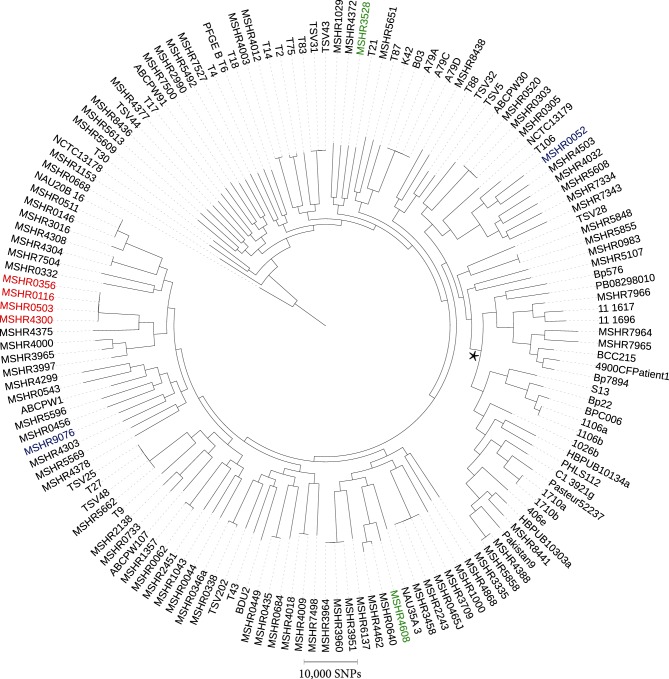
MP phylogeny of 150 *Burkholderia pseudomallei* genomes constructed using 207 694 orthologous, biallelic SNPs. MSHR1153 was used as a reference, and the tree was rooted with MSHR0668. Suspected cases of ST homoplasy are coloured as follows: red=ST-149 (MSHR0116, MSHR0356, MSHR0503 and MSHR4300); blue=ST-722 (MSHR0052 and MSHR9076); green=ST-804 (MSHR3528 and MSHR4608). The asterisk denotes the branchpoint for the non-Australian clade.

**Fig. 2.**
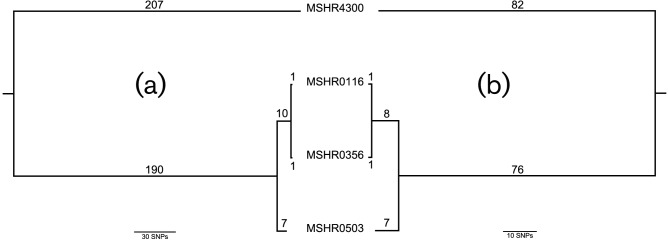
MP phylogenetic analysis of Australian ST-149 isolates, reconstructed using SNPs identified from comparative genomic analysis (reference genome: MSHR4300). (a) Based on 416 SNPs identified among the four ST-149 isolates, 404 SNPs separate the two environmental isolates, MSHR4300 and MSHR0503. (b) Post-Gubbins analysis to filter out recombinogenic SNPs shows an identical topology based on 175 SNPs, of which 165 SNPs separate MSHR4300 and MSHR0503. Consistency index for both trees=1.

We identified eight DPMS isolates belonging to three STs (ST-149, ST-722 and ST-804) as possible cases of intracontinental homoplasy, with isolates of these STs being separated by 460, 300 and 1000 km, respectively (Table 1). Based on a comparative analysis of 150 global genomes (Figs 1 and S1, available in the online Supplementary Material), geographically distant isolates belonging to ST-722 (MSHR0052 and MSHR9076) or ST-804 (MSHR3528 and MSHR4608) did not group closely together on the whole-genome level, differing by 21 211 and 20 567 SNPs, respectively. In contrast, the ST-149 environmental isolates MSHR0503 and MSHR4300 were separated by only 404 SNPs (Fig. 2a), which is comparable to previously observed intra-ST diversity [14]. Two clinical ST-149 isolates, MSHR0356 and MSHR0116, both of which were retrieved from melioidosis patients residing in a remote Australian island community where environmental isolate MSHR0503 was sampled [26], also grouped with MSHR4300, differing from this strain by 408 SNPs each. The three ST-149 isolates from the remote island community (MSHR0503, MSHR0356, MSHR0116) differed by a total of just 19 SNPs, suggesting very recent shared ancestry. When MSHR4300 was excluded and a draft assembly of MSHR0503 was used as the reference for read mapping, 18 SNPs were identified among these three strains, and only three SNPs separated the clinical isolates (MSHR0116 and MSHR0356) (Fig. S2).

**Table 1.** *Burkholderia pseudomallei* isolates used in this study

**Sequence type**	**Greatest linear distance** **between isolates (km)**	**Strain**	**Year**	**Sample type**	**Location**	**Genetic marker presence**		
						Cluster*	***bimA*†**	***fhaB*3**
149	460	MSHR0116	1992	Clinical	Remote island, Northern Territory	BTFC	Bp	Pos
		MSHR0356	1995	Clinical	Remote island, Northern Territory	BTFC	Bp	Pos
		MSHR0503	1997	Environmental	Remote island, Northern Territory	BTFC	Bp	Pos
		MSHR4300	2010	Environmental	Katherine region, Northern Territory	BTFC	Bp	Pos
722	300	MSHR0052	1989	Clinical	Darwin region, Northern Territory	YLF	Bp	Pos
		MSHR9076	1998	Clinical	Katherine region, Northern Territory	BTFC	Bm	Pos
804	1000	MSHR3528	2009	Environmental	Remote Central Australia, Northern Territory	BTFC	Bp	Pos
		MSHR4608	2011	Environmental	Darwin region, Northern Territory	BTFC	Bp	Pos

*BFTC, *Burkholderia thailandensis*-like flagella and chemotaxis cluster; YLF, *Yersinia*-like fimbriae cluster.

†Bp, *Burkholderia pseudomallei* type *bimA*; Bm, *Burkholderia mallei* type *bimA*.

Given that *B. pseudomallei* is highly recombinogenic, SNP differences separating the ST-149 strains could have arisen due to one or more recombination events, which can confound molecular clock analyses. We therefore assessed the contribution of recombination in the ST-149 isolates. In total, 416 biallelic SNPs were found among the four ST-149 isolates (Fig. 2a). Removal of the 241 recombinogenic SNPs did not affect the topology of the MP or ML phylogenies (Figs 2b and S3). Using an average substitution rate of 5.9×10^−7^ substitutions per site per year (95 % CI=2.0×10^−7^ to 9.8×10^−7^) [16, 27–29] and following the removal of recombinogenic SNPs, divergence of the two geographically disparate ST-149 isolates was estimated to have occurred in 1984 (95 % CI=1947 to 1992).

Finally, we investigated three variable genetic markers with known geographical [30] or clinical [31] associations in *B. pseudomallei*: the mutually exclusive *bimA*_Bp_ and *bimA*_Bm_ loci [32], the variably present virulence gene *fhaB3* [33], and the mutually exclusive *Burkholderia thailandensis-*like flagella and chemotaxis (BTFC) cluster and *Yersinia-*like fimbriae (YLF) cluster loci [30]. These genetic markers were developed in the pre-genomics era to provide a rapid determination of strain relatedness, virulence potential and origin [30, 31]. All isolates were positive for the virulence genes *fhaB*3 [33] and *bimA*_Bp_ [32], except for MSHR9076 (ST-722), which harboured the *B. mallei*-like neurotropic variant *bimA*_Bm_ [34] instead of *bimA*_Bp_. In addition, MSHR0052 (ST-722) was YLF-positive, whereas all other isolates possessed the BTFC cluster, including MSHR9076 (Table 1). These three markers were unable to differentiate the ST-804 strains; however, this result was not surprising as *bimA*_Bp_*, fhaB*3 and BTFC comprise the most common genotype in Australian isolates [30, 31]. The three variable genetic markers were also identical across the four ST-149 isolates, consistent with their close phylogenomic relatedness.

## Discussion

MLST remains a well-adopted genotyping method for bacterial epidemiological studies due to its robustness, portability and data accessibility [5]. Although MLST can provide a snapshot of the evolutionary history and relatedness of an isolate, there are certain instances where MLST does not perform as intended and may in fact provide misleading results, particularly for highly recombinogenic species such as the melioidosis bacterium, *B. pseudomallei* [35]. WGS has previously been used to resolve two such cases of ST homoplasy that occurred between Australian and Cambodian isolates of *B. pseudomallei* [13]. Here, we performed WGS analysis on eight Australian *B. pseudomallei* isolates from three STs (ST-149, ST-722 and ST-804) suspected of also being homoplastic due to shared STs despite wide geographical separation.

Phylogenomic analysis confirmed that the ST-722 and ST-804 isolates represent two new ST homoplasy cases, with isolates of each ST separated by >20 000 SNPs, although all still resided within the Australian clade (Figs 1 and S1). This genetic distance is characteristic of Australian isolates belonging to different STs. In addition, the two ST-722 isolates differed at the YLF/BTFC cluster and *bimA*_Bp/Bm_ loci, providing further evidence of their dissimilarity. Our results add to the two previously documented intercontinental ST homoplasy cases reported by De Smet *et al*. [13]. Although ST homoplasy cases are rare, they represent important instances of genotyping failure that can significantly confound analyses that rely on MLST data, particularly where accurate source attribution is paramount, such as in forensic or outbreak investigations. It is therefore crucial that high-resolution techniques such as WGS are used to confirm unusual or unexpected genotyping results, especially in highly recombinogenic pathogens such as *B. pseudomallei.*

Unlike ST-722 and ST-804, ST-149 isolates showed evidence of relatively recent diversification. Despite being retrieved from soil samples separated by 460 km, the environmental isolates differed by just 404 SNPs (Fig. 2a), or 165 non-recombinogenic SNPs (Fig. 2b). Using molecular clocks to calculate divergence has gained popularity in recent years. However, such estimates in *B. pseudomallei* have proven to be non-trivial due to uncertain generation times and difficulties in estimating the contribution of recombination to genetic diversity. Nevertheless, our results estimate that divergence of ST-149 occurred in 1984 (95 % CI=1947 to 1992). Based on this estimate, we hypothesise that an anthropogenic, zoogenic, ecological or severe weather event drove the long-range dispersal of ST-149 between these two locations sometime between 18 and 63 years before MSHR4300 was isolated in 2010.

The ST-149 case is not the first instance of long-range *B. pseudomallei* dispersal. A possible intracontinental long-range dispersal event is thought to have occurred in Western Australia across a linear distance of 500 km [36], although WGS would be needed to rule out ST homoplasy in this case. The largest known long-range dispersal event has recently been documented between Asia and Australia, with the unprecedented transmission of an Asian *B. pseudomallei* clone, ST-562, into Darwin, the capital city of the Northern Territory [14]. Although the precise origin and transmission of this clone into Australia has not yet been determined, its restricted geographical locale and very low levels of genomic diversity point to a very recent introduction event [14, 36]. Our current study adds to the growing body of work supporting the potential long-range transmission of *B. pseudomallei* across large geographical distances. The precise factors driving these dispersal events requires ongoing monitoring and investigation.

## Conclusion

We report, for the first time, two instances of intracontinental ST homoplasy in *B. pseudomallei*. These examples illustrate the occasional limits of MLST for accurate source attribution of highly recombinogenic species. We also report an unusual instance of long-range *B. pseudomallei* dispersal, which may have been aided by recent anthropogenic, ecological or weather-related factors.

## Data bibliography

1. **MSHR0503**
2. Sahl JW, Vazquez AJ, Hall CM *et al.* BioProject PRJNA285704, Sequence Read Archive Number SRR2975627 (2016) https://www.ncbi.nlm.nih.gov/sra/SRR2975627
3. **MSHR0356**
4. Nandi T, Holden MT, Didelot X, *et al.* BioProject PRJEB2866 European Nucleotide Archive ERR311049 (2015), https://www.ncbi.nlm.nih.gov/sra/ERX284330
5. **MSHR4300**
6. Johnson SL, Baker AL, Chain PS, *et al.* NCBI BioProject PRJNA230723 (2015) https://www.ncbi.nlm.nih.gov/Traces/wgs/?val=JPQI01
7. **MSHR1153**
8. Bunnell A, Chain PS, Chertkov O *et al.* NCBI BioProject PRJNA231860 (2015) https://www.ncbi.nlm.nih.gov/bioproject/231860

## Supplementary Data

506 Supplementary File 1
