# Supplementary material for: Suspected cases of intracontinental Burkholderia pseudomallei sequence type homoplasy resolved using whole-genome sequencing
Source: Microb Genom. 2017 Nov 14;3(11):e000139. doi: 10.1099/mgen.0.000139 (PMC5729916; doi:10.1099/mgen.0.000139)
Supplement: Supplementary File 1 [file mgen-3-139-s001.pdf]

SUPPLEMENTAL DATA

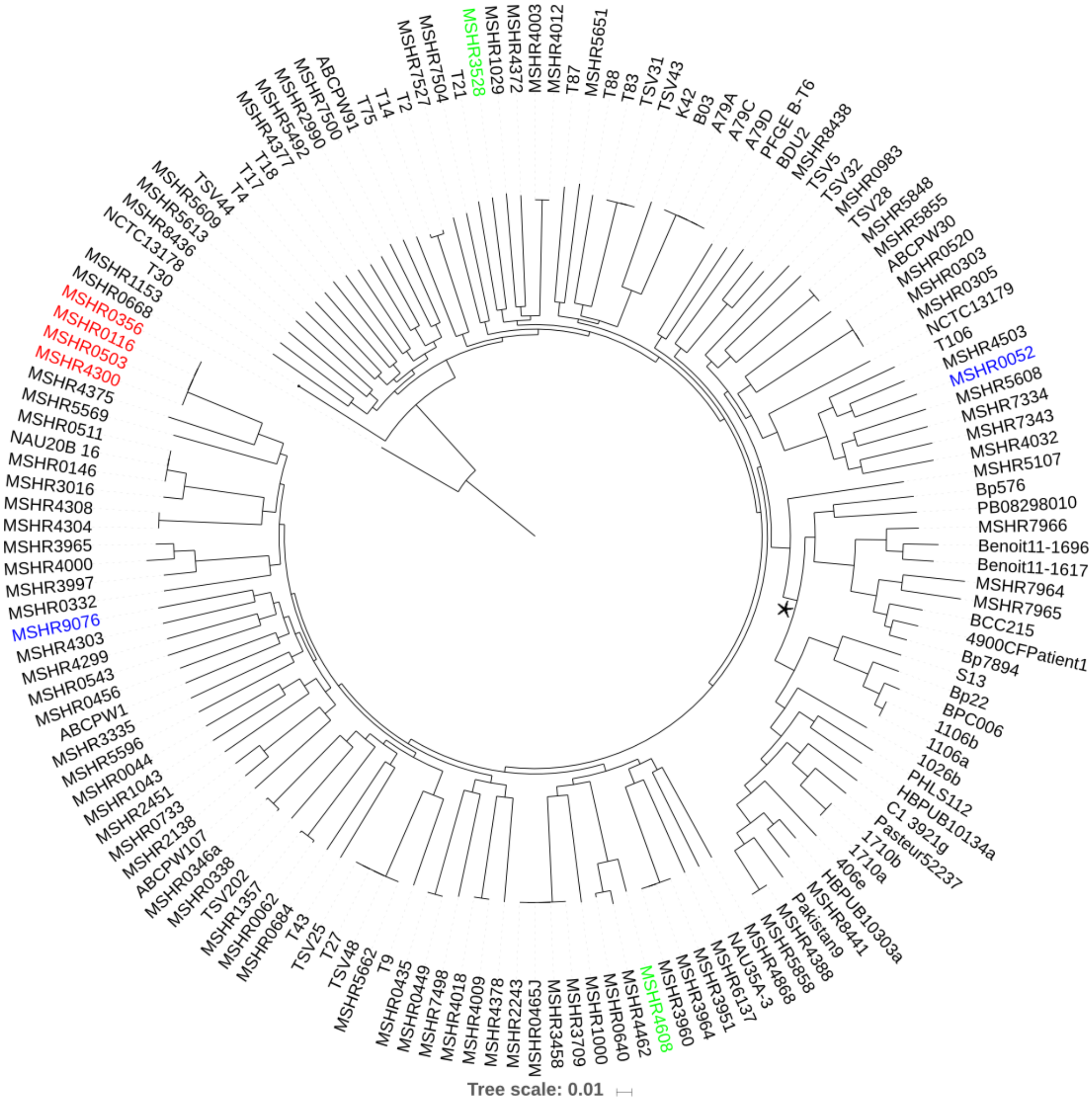

Figure S1. Maximum likelihood phylogenetic analysis of 150 *Burkholderia pseudomallei* genomes constructed using 207,694 orthologous, biallelic SNPs. MSHR1153 was used as a reference, and the tree was rooted using MSHR0668. Suspected cases of ST homoplasy are coloured as follows: red, ST-149 (MSHR0116, MSHR0356, MSHR0503, and MSHR4300); blue, ST-722 (MSHR0052 and MSHR9076); green, ST-804 (MSHR3528 and MSHR4608). The asterisk denotes the branch point for the non-Australian clade. The topology of this tree was highly similar to that of the maximum parsimony tree (Fig. 1).

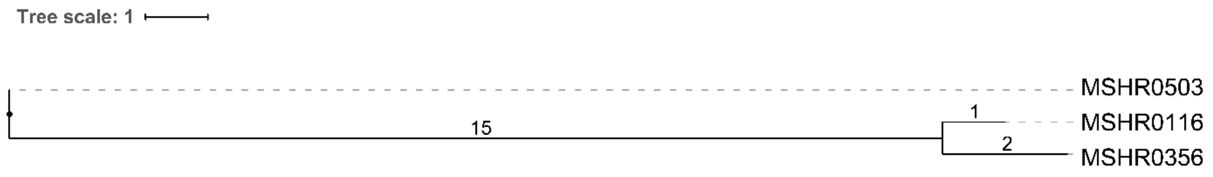

Figure S2. Maximum parsimony phylogenetic analysis of remote Australian island *Burkholderia pseudomallei* isolates belonging to ST-149, using the environmental isolate MSHR0503 as a reference genome. Eighteen SNPs were identified, with 15 SNPs separating the common ancestor of the clinical isolates (MSHR0356 and MSHR0116) from the environmental isolate (MSHR0503).

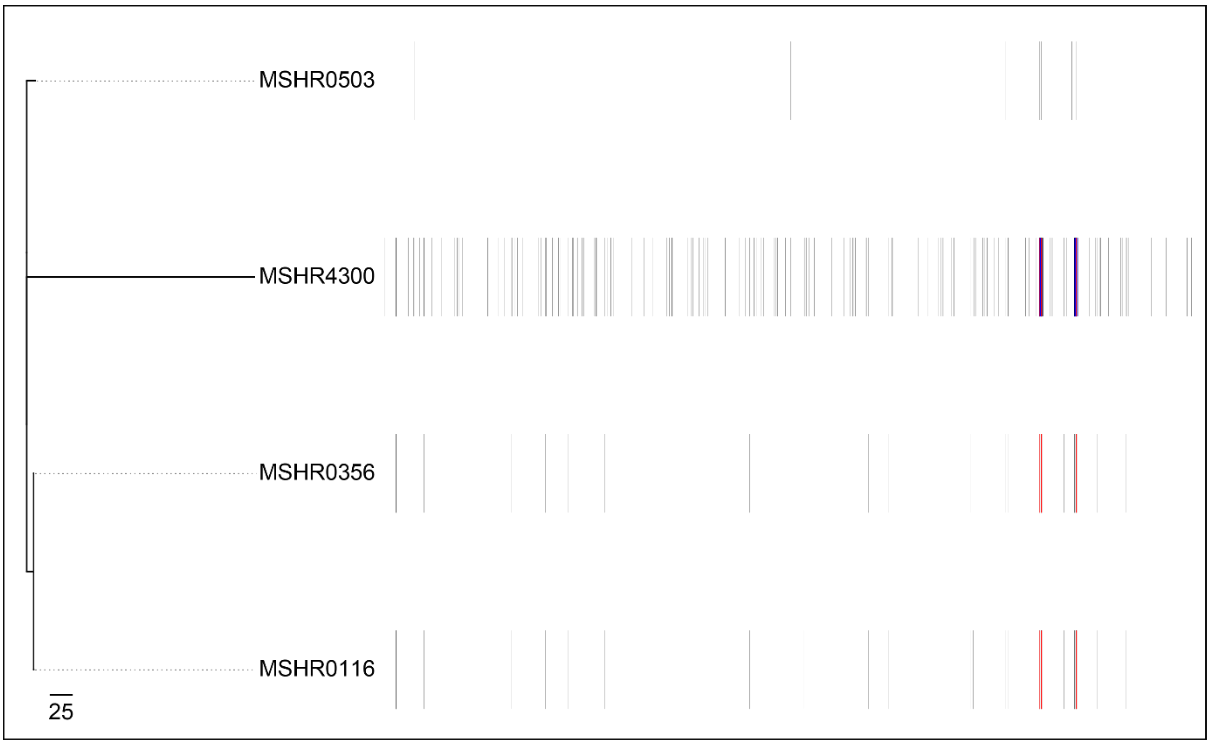

Figure S3. Phylogenetic reconstruction of ST-149 genomes, with predicted regions of recombination according to Gubbins [26]. Black bars: all SNPs; red and blue bars: synapomorphic and autapomorphic SNPs, respectively, identified in recombinogenic regions. SNPs found in recombinogenic regions were removed prior to molecular clock analysis.
